# Identification of Prodromal Parkinson Disease: We May Be Able to But Should We?

**DOI:** 10.1212/WNL.0000000000209394

**Published:** 2024-05-17

**Authors:** Richard N. Rees, Alastair J. Noyce, Anette E. Schrag

**Affiliations:** From the Department of Clinical and Movement Neuroscience (R.N.R., A.E.S.), UCL Queen Square Institute of Neurology, University College London; Centre for Preventive Neurology (A.J.N.) and Wolfson Institute of Population Health (A.J.N.), Queen Mary University of London; and Department of Neurology (R.N.R.), St George's University NHS Foundation Trust, London, UK.

## Abstract

Parkinson disease (PD) remains a progressive and incurable disease. Research over the past decade provides strong evidence of a detectible phase before the clinical diagnosis, known as the prodromal phase of PD (pPD). In this article, we review the debate about disclosure of risk of progression to PD and related disorders to individuals through the perspectives of the pillars of medical ethics: beneficence, nonmaleficence, autonomy, and justice. There is evidence that lifestyle modification may have positive effects on onset and progression of PD, providing justification of potential benefit. From a societal perspective, a diagnosis of pPD could allow targeted recruitment to disease-modifying trials. Regarding nonmaleficence, direct evidence that catastrophic reactions are scarce is largely derived from studies of monogenic conditions, which may not be generalizable. Diagnosis of PD can be traumatic, and appropriate communication and evaluation of circumstances to weigh up disclosure is crucial. Future research should therefore examine the potential harms of early and of false-positive diagnoses and specifically examine these matters in diverse populations. Autonomy balances the right to know and the right not to know, so an individualized patient-centered approach and shared decision-making is essential, acknowledging that knowledge of being in the prodromal phase could prolong autonomy in the longer term. Distributive justice brings focus toward health care and related planning at the individual and societal level and affects the search for disease modification in PD. We must acknowledge that waiting for established disease states is likely to be *too little, too late* and results in failures of expensive trials and wasted participant and researcher effort. Ultimately, clinicians must arrive at a decision with the patient that solicits and integrates patients' goals, taking into account their individual life circumstances, perspectives, and philosophies, recognizing that one size cannot fit all.

## Does Prodromal Parkinson Disease Exist, and Does It Matter?

In recent years, there is a growing literature on identifying individuals who may be in an early, prodromal, or prediagnostic phase of Parkinson disease (PD). Efforts have been made to identify people from specific risk cohorts, be that monogenic subtypes of PD, such as *SNCA*,^1^ *LRRK2*,^2^ and *GBA*,^3^ or from cohorts of patients with isolated REM sleep behavioral disorder: a synucleinopathy with very high rates of conversion to parkinsonism.^4^ However, there is acknowledgment that not only do these specific at-risk groups not represent most people who develop *idiopathic* PD, there may also be differences in both established and prodromal phenotypes, all likely underpinned by differences in the pathobiology of different subtypes of PD. Therefore, several attempts have been made to review the general population and identify those at risk through prediction models based on known risk and protective factors with or without additional prodromal symptoms or markers, such as hyposmia, enlarged areas of nigral hyperechogenicity using transcranial sonography, mood disorders, or combinations of these.^5,6^ These efforts will now likely be further reinforced by the recent development of highly accurate CSF (and potentially serum) α-synuclein seed amplification assays (SAA), which may show the signature of disease before diagnostic signs are overt.^7^

More than merely an intellectual exercise, what is the purpose of being able to identify patients in the prodromal phase? First, there is an increasing acknowledgment that PD, as currently defined, is unlikely to be one homogeneous condition because significant heterogeneity is seen not only in presentation but also in progression, severity, total symptom burden, and treatment response (and side effects).^e4^ This heterogeneity is not just born out clinically but is also seen pathologically.^8^ Identification of the earliest stages and then being able to determine phenotypic differences may then lead to better understanding of the first phases of the pathobiology, and ultimately to the creation of better, more targeted, more personalized treatments.

The second argument is that by the time patients enter the final common pathway that is termed *Parkinson disease*, the pathologic cascade is likely to be irreversible and may be unstoppable. Given that at (motor) diagnosis, 50% of dopaminergic neurons have been lost,^9^ it follows that some method of arresting the disease before this point is necessary. Treatments that slow, stop, or reverse the pathologic process, rather than merely treating the symptoms, are known as disease-modifying therapies (DMT).^e5^ While not proven, it may be that DMTs that are effective in monogenic PD could also be effective in idiopathic PD.^e5,e6^

## The Ethics of Prodromal Diagnosis

The biomedical model of disease is based on mechanistic understanding of the origin and progression of disease. Great progress has been made to survival in the worlds of oncology and infectious disease based on this understanding and gives credence to the axiom that *early detection is better*. Within these contexts, there are treatments (e.g., antibiotics) that stop the development of sepsis and death, or less aggressive treatments can be given to local compared with metastatic disease (with screening programs designed for this reason). In these contexts, the natural history of the conditions is well understood and survival curves well mapped out, with and without treatment. In neurology, we have seen that immunotherapy after a first clinically isolated syndrome in multiple sclerosis changes the risk of progressive disease and disability.^10^ In neurodegenerative processes, however, this axiom may not necessarily hold true, with much longer disease trajectories, greater heterogeneity of progression, less certainty that the condition will become manifest within the patient's lifetime, and importantly, a dearth of treatments that significantly alter the natural history. For each individual therefore, the focus may not be on *early* diagnosis, but on *timely* diagnosis.^11^

Assuming that a robust diagnosis of prodromal PD can be made, arguments for and against this can be identified by referring to 4 of the timeless pillars of medical ethics—beneficence, nonmaleficence, autonomy, and justice.^12^

## Beneficence

The argument of beneficence (i.e., providing benefits balancing against risks and costs) holds that if a person's destination is to have PD, the earlier they know about it, the earlier they can take steps to ameliorate it by whatever means are available.

Currently, there are no DMTs available; however, that is not to say that methods have not been identified that do have evidence of positively affecting disease course. Aerobic exercise is associated with reduced risk of PD with an OR of 0.67 in meta-analyses^13^ and may well positively affect disease progression through its benefits on mood, cortical atrophy, cardiovascular function, bone health, or even through increasing levels of neuroprotective cytokines and growth factors (such as GDNF, which itself has been used exogenously in trials for neuroprotection).^14^ There is evidence that balance and mindfulness-based exercise techniques such as tai-chi or qi-gong can have considerable effect on motor function, depression, and quality of life in PD.^15^ This goes further than the general advice for healthy living including regular exercise and good diet: pairing this specific advice with the threat of a neurodegenerative disease overcomes the natural discounting of personal risk and can lead to lasting and meaningful behavioral change.^16^

Furthermore, in the age of multimorbidity, people with PD can be empowered to take an active role in managing other conditions (for which there may be crossover regarding both treatment and effect). It has been shown that recently diagnosed patients with PD have high and undertreated cardiovascular risk, associated with worse motor and cognitive phenotype.^17^ There may also be overlap with treatment, given the recent studies of statins for possible neuroprotective (and definitively cardioprotective) properties (although so far this has not resulted in any new DMTs after the negative results of the PD-STAT study).^18^

Earlier knowledge of diagnosis can also lead to earlier development of support, both formal and informal, and understanding that some symptoms may be directly attributable to the condition rather than misplaced attribution to aging or alternative diagnoses: in turn leading to evidence-based treatments and improved quality of life.

Within the PREDICT-PD study, we have shown motor dysfunction that is clinically evident,^19^ and others have found similar degrees of dysfunction in other prodromal cohorts. The ELLDOPA trial showed that there were no benefits to delaying dopamine replacement therapy,^20^ and Grosset and colleagues have shown that quality of life is worse in patients who elected to delay dopamine replacement therapy.^21^ It therefore stands to reason (and would be worthy of dedicated study) that people in the prediagnostic phase who have motor impairment may also benefit from levodopa, without risking longer-term efficacy as the disease progresses.

From a societal perspective, an argument of beneficence could be applied even today, given that even moderately robust identification of people in the prodromal stage could allow for recruitment into trials of disease-modifying drugs.^22^ This would avert a potential problem that has blighted the search for a “cure” for Alzheimer disease, whereby potentially viable products are being abandoned after failed studies in people with advanced or even mild-to-moderate disease stages—a problem that has been summed up as *too little, too late*.^23^

## Nonmaleficence

Regarding nonmaleficence—*do no harm*—a strong argument can be made for not providing a diagnosis in the prodromal stage. This holds particularly true while there is an absence of DMT. A diagnosis of an incurable, progressive condition could cause serious social and psychological harm. Schaeffer and colleagues therefore surveyed 101 patients with established PD about their views on prediagnostic risk disclosure. Approximately 54% of respondents would not have wanted to know their risk in the absence of DMT, yet 85% would have wanted to know if they felt that they could apply lifestyle changes that might modify the disease course.^24^ Of them, 90% of patients with RBD would want information on the link with progressive neurologic conditions, with only 2/31 patients not wanting the information due to a feeling that nothing can be done.^25^ Receiving a diagnosis of PD in those seeking a diagnosis for their symptoms can be a traumatic event, particularly when not communicated well,^26^ but the actual evidence for causing harm with a prodromal diagnosis is not strong. In a review of quality of life after predictive testing or early identification of neurodegenerative disease (largely focusing on predominantly genetic conditions), Paulsen and colleagues found that extreme or catastrophic outcomes are rare. However, there were side effects to disclosure of risk including transiently increased anxiety and/or depression but that most participants report no regret.^27^

An area that has not been investigated is the potential harm caused by erroneous diagnosis. A clinical diagnosis of PD by movement disorder experts is only accurate in 82% of cases; while in a recent review by Bellomo et al.^28^ of SAA techniques in PD range from 80% to 100% (albeit using different gold standards). A diagnosis of prodromal PD that rests on the diagnosis of a related condition such as RBD is further complicated given that this can also be present in other neurodegenerative conditions, not just PD. This suggests that even with the best available assessment, there is a reasonable risk of a false diagnosis of prodromal PD, the harms of which have not been explored.

## Autonomy

Autonomy in relation to risk disclosure can be summed up as the right to know and the right not to know.^29^ However, as highlighted by Dhedhi et al.,^30^ there are 2 actors in the process of a diagnosis: the clinician and the patient. Good communication and medical practice attuned to the patient and shared-decision making would include ascertaining what the patient's wishes were in receiving such a diagnosis. This approach was supported by a minority of neurology/neuroscience experts surveyed on their perspectives on disclosing a diagnosis: 29% reported that they would only disclose risk of PD under certain circumstances, including that the patient had specifically asked for such disclosure, while 12% would *never* disclose someone's (calculated) risk of PD if there was not a concurrent diagnosis of RBD.^31^ Unlike offering a diagnosis of mild cognitive impairment (MCI) in the context of a primary dementing process, and the specter of Alzheimer disease (AD) or dementia weighing heavy on the mind of the patient,^32^ prodromal PD carries with it an inevitability of progression that MCI specifically avoids. With that lack of ambiguity (the explicit understanding that MCI represents a stage of some overlap between the effect on memory of normal aging and an active and progressive dementing process, from which only some patients will progress), a sense of helplessness and loss of agency could develop, which would then undermine some of the potential benefits outlined earlier. Although the historic lack of fluid biomarkers has hampered early diagnostic certainty, there is growing evidence of the accuracy of SAA, not only in established PD but also in prodromal states.^28^ This could create space for a term such as at risk of Parkinson disease.

Considering the concept of autonomy from a different perspective, an early diagnosis of a neurodegenerative disease that may rob a patient of their capacity to make informed decisions (not only in matters of health and health care but also regarding finances and other complex life decisions), early diagnosis can be a prompt to discussing with professionals and with family legal constructs to safeguard independence and decision-making, such as Lasting Powers of Attorney and Wills, which have been identified as an important but not well understood or considered aspect of life by UK patients with PD.^33^ While it could be argued that these should be routine issues that all older adults should address, there is evidence that receiving a diagnosis of a neurodegenerative condition is a crucial prompt for behavioral change.^34^

## Justice

The fourth pillar of ethics is justice and is best considered in the light of distributive justice—the appropriate distribution of health care resources. In a recent estimate, the average direct cost of PD was $9924 (SD, $22,140)/person/year. While more than 50% of increased PD-related expenditure is incurred by those in the most advanced stages,^35^ disease modification would lead to reduction in the likelihood of reaching the advanced stage, and even early-stage PD has associated costs, related to not just to motor but also nonmotor features. For instance, in a review, a participant who was self-employed and installed electrical equipment in cars found that things that “previously I would've done say in a matter of a few seconds. It would take me several minutes to do ... You've got a limited amount of time to do it, so it increased the pressure on me as well. As a result the business was starting to suffer. I ended up having to sell my house because I couldn't afford the mortgage.” On an individual level, this is life-changing, and on a societal level, for a condition that affects up to 428/100,000 people in the last few years before the UK statutory retirement age (currently 66 years of age),^e10^ this represents a significant impact on the wider economy. Identification of people in the prodromal stage not only allows them to modify their own progression (and therefore reduce costs over the whole of their lifetime) but also allows for better, more cost-efficient planning of resources within the health and care sectors.

The principle of justice can also be appreciated by considering the potential ramifications of a diagnosis of prodromal PD outside the scope of health care. Driving a car is an activity that is closely tied with independence, autonomy, and quality of life. For many people outside large cities with good public transport, and for most in North America, driving is almost essential for all work, social, and leisure activities outside the home. PD can affect the ability to drive safely due to motor, cognitive, and treatment effects.^e11^ In the United Kingdom, there is a legal requirement to inform the DVLA (Driver and Vehicle Licensing Agency) of a diagnosis of Parkinson disease, even if there is no immediate requirement to surrender one's license. This can bring stigma and fear of loss of independence and even how people feel competent to continue in employment.^36^ Closely tied to licensing and road safety is insurance. A diagnosis of PD can bring significant increases in the cost of premiums, not just to car insurance but to life, health, and travel insurance.^33^ These increased costs reflect the actuarial risks, based on the data available for established PD, disease progression, and mortality. However, these assumptions will not hold true when motor and nonmotor signs are significantly milder, yet the risk might not be adjusted in financial markets. Therefore, receiving a diagnosis of prodromal PD could affect significant life and lifestyle choices. There would need to be safeguarding against discriminatory practices, which would need collaboration between medical researchers, patient bodies and charities, the legal and financial systems, and governmental stakeholders. This ties in with another of the themes outlined by Paulsen and colleagues, in which “stigmatization, discrimination, and environmental effects are poorly understood ... and further policy and laws are required to protect consumers.”^27^

A final compelling argument of justice is that of clinical trials. The *holy grail* in PD research is finding an effective DMT: something that halts or reduces the rate of progression and therefore allows patients to retain independence, quality, and quantity of life.^e5^ As the needle is starting to move for AD with recent approval of potentially effective DMTs, after many decades and billions of dollars of drug development and trials, the PD community can learn many lessons from that journey. Treatment failure is multifactorial, with flaws in target identification, drug delivery, target engagement, assessment of change, and disease heterogeneity: these problems have some generic transferability to PD, but the detail is specific to the disease. On the other hand one frequently described problem is that treatments are being tested in populations of patients with advanced, or at least established disease, and therefore, trials in these populations represent the problem of being *too little, too late*.^23^ Identifying people in the prodromal phase, with evidence of protein aggregation from seed amplification assays, potential treatments can be given to smaller numbers of participants. This will reduce the cost of these studies and by excluding healthy older adults with non-neurodegenerative causes of symptoms of aging may decrease the risk of rejecting viable treatments.^37,38^ This can then be further augmented by using seed amplification assays to confirm the presence of alpha-synuclein for a truly enriched population with evidence of protein aggregation and subtle motor and nonmotor clinical manifestations.

At the crossroads of beneficence, nonmaleficence, and justice is the consideration of why someone diagnosed with prodromal Parkinson disease might volunteer for trials. Olsen and colleagues investigated this in the context of PD and found a complex interplay between altruism and self-interest based on potential benefit and harm avoidance.^e12^ Patients with RBD have explicitly stated that they would want to know the link with neurodegenerative diseases because this would allow trial participation.^25^ The potential to add meaning to the lives of people through trial participation could be seen as another argument, although this should not be overstated.

The concept of justice also forces providers to think about the challenges faced by different populations and cultures. Recent data suggest that non-White people with PD have worse health-related quality of life, even when under the care of a specialist center.^39^ There are also widespread differences in access to health care, utilization of specialist services and drugs, clinical trial participation, and new technologies or treatments in different global, regional, and national settings.^40^ It is essential that as the field progresses, specific research is undertaken to understand the impact on diverse populations. Furthermore, action needs to be taken at a policy and delivery level to address the unequal representation of minorities in clinical trials to conduct studies outside of the majority White, highly developed countries. To date, the research conducted to understand patient/public and physician attitudes to this topic have occurred solely in Europe, leaving out the voices of most people affected by PD.

## Conclusion

Ultimately, each of the arguments outlined earlier will be weighted differently across the population, and there cannot be a one-size-fits-all approach. We may be nearer to being able to diagnose PD at an earlier stage, based on mechanistic understanding of the progression of the disease and new biomarkers. Further work is vital to understand how these challenging consultations should be conducted, by whom (primary care physician, general neurologist, movement disorders specialist, and dedicated counsellors akin to genetic services), and at what pace, particularly because signposting toward this discussion may inadvertently let the cat out of the bag. Making a *timely* diagnosis requires the clinician to consider each of the ethical principles outlined and come to a mutual understanding with the patient, incorporating their understanding of the condition (and the likely progression of it), their goals, their fears, potential benefits, and possible harms (including biological, psychological, and social).^e13^ Of importance, communication of the diagnosis of pPD, with an adequate explanation of the uncertainties and potential implications and consequences and follow-up for questions would be a prerequisite of any such disclosure.

As we refine our understanding and diagnostic ability in the prodromal phase of PD, the debate needs to move beyond the scientific community, and engagement in the public and political spheres is needed. The promise of DMTs for PD is no longer over the horizon, and a number of trials with potentially disease-modifying agents are currently ongoing. By this point, we need to have understood what the prodromal phase means not only in quantitative and mechanistic terms but also what such a diagnosis might mean in the context of a patient's social, cultural, ethical, and health system backgrounds.

## Appendix Authors

**Table:**

Name	Location	Contribution
Richard N. Rees, MBChB, MD(Research), MRCP	Department of Clinical and Movement Neuroscience, UCL Queen Square Institute of Neurology, University College London; Department of Neurology, St George's University NHS Foundation Trust, London, UK	Drafting/revision of the article for content, including medical writing for content; major role in the acquisition of data; study concept or design; and analysis or interpretation of data
Alastair J. Noyce, MBBS, BSc, MRCP	Centre for Preventive Neurology and Wolfson Institute of Population Health, Queen Mary University of London, UK	Drafting/revision of the article for content, including medical writing for content; analysis or interpretation of data
Anette E. Schrag, MD	Department of Clinical and Movement Neuroscience, UCL Queen Square Institute of Neurology, University College London, UK	Drafting/revision of the article for content, including medical writing for content; analysis or interpretation of data

## Glossary

AD: Alzheimer disease
DMT: disease-modifying therapies
MCI: mild cognitive impairment
PD: Parkinson disease
SAA: seed amplification assays
